# Cardiovascular magnetic resonance in carotid atherosclerotic disease

**DOI:** 10.1186/1532-429X-11-53

**Published:** 2009-12-15

**Authors:** Li Dong, William S Kerwin, Marina S Ferguson, Rui Li, Jinnan Wang, Huijun Chen, Gador Canton, Thomas S Hatsukami, Chun Yuan

**Affiliations:** 1 Department of Radiology, University of Washington, Seattle, WA, USA; 2 Clinical Sites Research Program, Philips Research North America, Briarcliff Manor, NY, USA; 3 Department of Surgery, University of Washington, Seattle, WA, USA

## Abstract

Atherosclerosis is a chronic, progressive, inflammatory disease affecting many vascular beds. Disease progression leads to acute cardiovascular events such as myocardial infarction, stroke and death. The diseased carotid alone is responsible for one third of the 700,000 new or recurrent strokes occurring yearly in the United States. Imaging plays an important role in the management of atherosclerosis, and cardiovascular magnetic resonance (CMR) of the carotid vessel wall is one promising modality in the evaluation of patients with carotid atherosclerotic disease. Advances in carotid vessel wall CMR allow comprehensive assessment of morphology inside the wall, contributing substantial disease-specific information beyond luminal stenosis. Although carotid vessel wall CMR has not been widely used to screen for carotid atherosclerotic disease, many trials support its potential for this indication. This review summarizes the current state of knowledge regarding carotid vessel wall CMR and its potential clinical application for management of carotid atherosclerotic disease.

## Introduction

The common carotid artery bifurcation is a zone of frequent atherosclerotic disease owing to the disturbed hemodynamics in the carotid bulb. Estimates place carotid atherosclerosis as the embolic source behind up to 30% of the 700,000 new or recurrent strokes annually in the United States [1]. With 5.8 million stroke survivors in the U.S., stroke is the leading cause of long-term disability and results in direct and indirect costs of $68.9 billion per year [2]. As a common source of stroke, carotid atherosclerosis contributes substantially to the overall healthcare burden and costs both in the U.S. and worldwide.

To assess the risk of stroke from carotid atherosclerotic disease, the current standard is to measure the severity of luminal stenosis by angiography. Both the European Carotid Surgery Trial (ECST) and the North American Symptomatic Carotid Endarterectomy Trial (NASCET) showed that individuals with prior ischemic symptoms and carotid stenosis in excess of 50% were at very high risk of stroke and that the risk was significantly reduced by surgery [3,4]. In asymptomatic individuals, studies are more equivocal and the benefit of surgery or screening for asymptomatic stenosis is hotly debated [5-8]. Notably, asymptomatic carotid stenosis in excess of 50% is very common with prevalence estimated at 12.5% in men and 6.9% in women over age 70 [9].

Furthermore, patients with high-grade stenosis likely represent just a fraction of those at risk of stoke due to carotid atherosclerosis. Given the unique geometry of the carotid bulb, which generally is larger in diameter than the more distal internal carotid, it is possible to have a minimal stenosis despite the presence of significant plaque burden. Furthermore, luminal stenosis measurement may underestimate plaque burden because of compensatory, expansive arterial remodeling, as originally described by Glagov [10]. Consequently, large advanced plaques are commonly observed in carotid arteries without measurable stenosis [11] and have been implicated in thromboembolic complications [12].

Given the uncertainty in patient management for high-grade asymptomatic stensosis and the failure of stenosis to characterize risk from lesions that do not compromise the lumen, better strategies for risk assessment are needed. Cardiovascular magnetic resonance (CMR) of the carotid vessel wall provides a new imaging approach with potential to identify the characteristics of the carotid atherosclerotic lesion itself. Carotid vessel wall CMR uses a combination of bright-blood and black-blood techniques to provide information regarding the structure of the carotid artery and the composition of the atherosclerotic plaque. These structural and compositional features are thought to play the central role in determining the risk posed by a carotid lesion. Thus, carotid vessel wall CMR may be the much needed tool for determining risk above and beyond stenosis.

The potential for direct and indirect clinical impact of carotid vessel wall CMR is not, however, limited to near-term stroke prediction. The ideal therapeutic approach would prevent the high-risk lesion from ever developing. In this regard, carotid CMR has the potential to evaluate the performance of therapies in clinical trials, and to identify new targets for therapy to prevent progression toward high-risk lesions. Carotid CMR may also play a role in selecting and monitoring optimal therapy for specific lesion characteristics. Finally, the carotid artery can be used as a systemic marker of overall cardiovascular health to be assessed by carotid CMR.

To understand the many roles that carotid CMR can play in clinical management of carotid atherosclerosis, this review describes the near-term feasibility and capability of carotid vessel wall CMR. By considering how carotid atherosclerotic tissues appear on CMR, image interpretation criteria are provided for the key targets. We examine the carotid atherosclerotic plaque in various stages from the already disrupted plaque back to the early stage of plaque formation to see how CMR can play a role in each stage. The direct and potential clinical impacts of carotid vessel wall CMR are addressed.

### Feasibility of Clinical Carotid vessel wall CMR

The attractiveness of CMR for assessment of the carotid artery wall derives from several factors. From a general imaging perspective, the carotid artery has beneficial physiology given its relatively large size, superficial location and remoteness from heart and lung motion. Phased-array surface coils designed for carotid artery provide sufficient signal-to-noise ratio (SNR) for the high-resolution imaging required for characterizing plaque composition [13,14]. CMR itself comes with the standard benefits of being non-invasive and avoiding the use of ionizing radiation, which is particularly important for serial imaging studies.

The foremost advantage of CMR for this application is the ability to combine information from multiple contrast weightings. Through early investigations, a standardized carotid vessel wall CMR protocol emerged consisting of black-blood fast spin echo sequences including T1-weighted (T1W), T2-weighted (T2W), and proton density weighted (PDW) imaging with fat suppression, combined with a bright-blood time of flight (TOF) sequence [15]. More recently, highly T1-weighted gradient echo sequences such as Magnetization-Prepared Rapid Acquisition Gradient-Echo (MP-RAGE) have been incorporated into carotid protocols [16]. Furthermore, these techniques can be augmented with injected contrast agents that provide improved or complementary information regarding plaque composition [17-19].

The availability of carotid plaque specimens from carotid endarterectomy has enabled the contrast features of plaque components to be validated and described in detail [20]. These studies have utilized serial histological cross sections with specialized staining to compare with the contrast features in corresponding cross-sectional CMR images acquired *in vivo*. Characteristics of common plaque components on CMR are summarized in Table 1. The optimal combination of contrast weightings depends on the imaging target(s) and will be discussed at length in the subsequent sections of this review.

**Table 1:** Carotid CMR Characteristics

	TOF	T1W	T2W	PD	CE-T1W*
**Fibrous tissue**	o	o	o	o	o
**Calcification**	-	-	-	-	-
**Lipid-Rich Necrotic Core**	o	o	-	-/o	-
**Hemorrhage**	+	+	-/+	-/+	-

Note: + indicates hyper-intense; o indicates iso-intense; - indicates hypo-intense

*Contrast enhanced T1W (CE-T1W): + indicates enhancement present; - indicates enhancement absent

### Technical considerations

Perhaps the greatest challenge for carotid wall CMR is the resolution to distinguish the internal plaque structure. Even in advanced disease, the vessel wall may only measure a few millimeters in thickness, with the potential existence of multiple substructures across that dimension. These structures also change shape rapidly in the longitudinal direction of the artery. Thus, 2D imaging approaches have had to contend with in-plane resolutions of 0.6 mm or less and slice thickness of 2-3 mm. Recently, 3D isotropic imaging approaches have been proposed with isotropic resolution up to 0.6 mm [21]. In both 2D and 3D approaches, this resolution pushes the SNR limits of the scanner, requiring long acquisition times. Minimizing patient motion during the scan with the use of comfortable head holders or motion-corrected scanning is critical. Also, because of SNR limitations, the use of parallel imaging to reduce scan time is not recommended.

The increasing availability of 3.0 Tesla field-strength scanners provides some SNR benefits for carotid vessel wall CMR. Compared to 1.5 T CMR, black-blood carotid artery MR images at 3.0 Tesla have been found to have 1.4-2.4 times increased carotid wall SNR and lumen/wall contrast-to-noise ratio [22-24]. The higher SNR can be traded for reduced scan time of up to about 40-50% [22].

The novel developments of coils, sequences, and scanner, taken together, are a driving force for carotid vessel wall CMR to obtain higher spatial resolution within a reasonable scanning time on a clinically available scanner.

### Image postprocessing

The importance of quantification in evaluating plaque burden and composition suggests a role for image postprocessing techniques. Highly automated measurement techniques can reduce analysis time, reduce reader-dependent bias, and improve measurement reproducibility. Several semi-automated imaging processing tools have been used to quantitative indices of plaque burden and composition [25-27]. Underhill et al. proposed a technique using active shape models to segment the vessel boundaries [28]. Utilizing morphologic information such as local wall thickness, Liu et al. proposed a flexible, multi-contrast plaque segmentation technique (Morphology-Enhanced Probabilistic Plaque Segmentation, MEPPS) that is suitable for measuring plaque composition *in vivo *[27]. Recently, Kerwin et al. described a plaque analysis software (The Computer-Aided System for Cardiovascular Disease Evaluation, CASCADE) to provide quantitative information regarding plaque burden and composition, which was highly similar to manual outlining but with considerably less analysis time [29].

### CMR appearance of Carotid Plaque

#### Disrupted plaque

**Figure 1:**
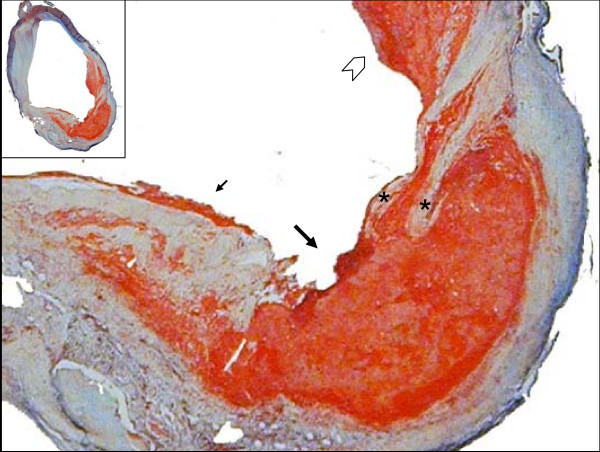
**The Ruptured Carotid Plaque**. A Movat's Pentachrome stain of a common carotid fibrous cap disruption. The disruption (big arrow) is framed by two branches of the fibrous cap (asterisks) on the right side, and a cap overlaid with thrombus on the left (small arrow). The chevron points to a region of organizing thrombus. (Magnification 100×, inset 10×)

For CMR, the most important clinical question to be answered for patients with established carotid lesions is whether the lesion poses a high, near-term risk for complications. Retrospective histological studies of carotid atherosclerotic lesions implicated in ischemic stroke and transient ischemic attacks have found those lesions to exhibit plaque disruption, most commonly fibrous cap rupture (Figure 1). Spagnoli et al. reported ipsilateral fibrous cap rupture was observed in 66.7% (64/96) of patients with major stroke, 23.1% (21/91) of patients with transient ischemic attack, and only 13.4% (11/82) of patients without prior ischemic symptoms [30]. Similarly, Carr et al. found that cap rupture occurred more frequently in symptomatic patients (74%) than in asymptomatic patients (32%) [31]. Once an atherosclerotic plaque is disrupted, the thrombogenic contents and lipid core are exposed to platelets and coagulation factors in circulating blood, predisposing the patient to an acute thromboembolic event. The study by Spagnoli et al. found that an acute thrombus was associated with cap rupture in 90.1% (64/71) patients with stroke. The clinical significance of plaque disruption begs the question whether CMR can visualize cap rupture. Of note, fibrous cap rupture and thrombosis can also occur in the carotid artery without overt clinical symptoms. Prior sites of rupture and thrombus can act as further stimuli for thrombus development. Thus, identification of these features on carotid vessel wall CMR could be an important part of a risk reduction strategy of stroke.

**Figure 2:**
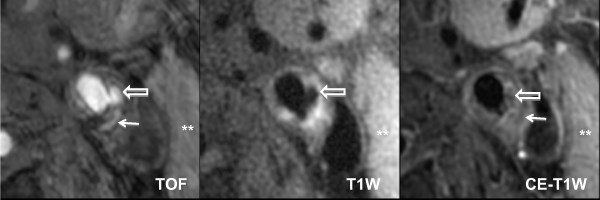
**Magnetic Resonance Images of the Ruptured Carotid Plaque**. The bright-blood 3D TOF image shows a distinct disruption of the fibrous cap (block arrow), corresponding to the hypointense juxtaluminal region on T1W and CE-T1W images. The hyperintense signal present in the necrotic core adjacent to the rupture (arrow) indicates presence of intraplaque hemorrhage inside of a lipid-rich necrotic core (thin arrow on CE-T1W image). Asterisks are placed on the sternocleidomastoid muscle, which is used to determine plaque signal intensities. The scan was acquired on a 3.0-T GE Signa Excite along with the use of a 4-element phased-array carotid coil.

To visualize plaque disruption and thrombus, black-blood CMR sequences (T1W, T2W or PD) permit a clear differentiation of both the lumen and outer wall boundaries by suppressing the signal from flowing blood. Plaque disruption can be identified by an ulceration of the plaque surface, the discontinuity of fibrous cap, and thrombus on the surface (Figure 2). In a histology-validated study of 26 patients scheduled for endarterectomy, Kampschulte et al. was able to detect luminal and juxtaluminal thrombus with an accuracy of 96%, by using an *in vivo *multi-contrast approach that primarily identified hyper-intense signals on TOF and T1W images that appeared to have direct contact with the lumen [32]. Moody et al. also used a non contrast-enhanced CMR method based on the T1-shortening properties of methemoglobin combined with a highly T1-weighted gradient echo protocol ("direct thrombus imaging") to identify hyper-intense regions of thrombus in the carotid artery [33].

In addition, black-blood sequences combined with bright-blood imaging techniques (3-dimensional TOF) can distinguish intact thick cap from intact thin and disrupted cap based on the lumen surface condition and the presence of a dark band along the lumen surface in TOF images [34]. The addition of gadolinium contrast agents, which lead to increase fibrous cap signal on T1W images, further aids in reliable assessment of fibrous cap status. A multi-contrast weighted CMR study including contrast enhanced images by kwee et al., found good intra- and inter-reader agreement (*k *= 0.96 for intra-reader; *k *= 0.64 for inter-reader) for fibrous cap status [35].

#### Thin Fibrous Cap and Lipid-Rich Necrotic Core (LRNC)

**Figure 3:**
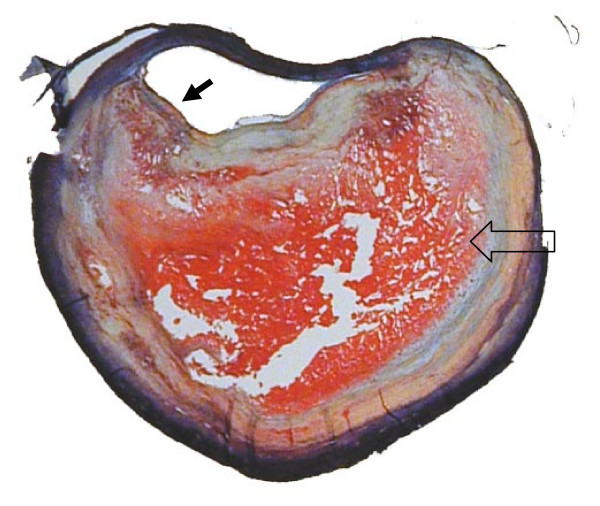
**The rupture prone atherosclerotic plaque has a thin fibrous cap overlaying a large necrotic core**. This Movat's Pentachrome stain shows a carotid plaque with a large necrotic core (block arrow) containing hemorrhage (red staining). The fibrous cap shows a dramatic thinning near the shoulder of the core (arrow in the lumen). (Magnification 10×)

While detection of prior plaque rupture has clinical implications, a more compelling question is whether detectable precursors of cap rupture exist. The concept of the disruption-prone or high-risk/vulnerable plaque, initially derived from coronary studies, has been increasingly shown to apply in the carotid circulation. Histological studies have shown that the most common feature of plaques prone to rupture is a thin fibrous cap with a large underlying necrotic core (Figure 3) [36-39]. These features are key targets of carotid vessel wall CMR and are potential imaging markers for risk of stroke/transient ischemic attack.

The well established results in the visualization of lipid-rich necrotic core (LRNC) have come from the use of black blood multi-contrast images (T1W, PDW, and T2W) using criteria that compares the signal intensity of the adjacent sternocleidomastoid muscle to the signal intensities of the black blood sequences [40-42]. The LRNC is a mixture of lipid, cellular debris, blood and water in various concentrations therefore MR signal intensities of the different sequences reflect those variations. In general, the LRNC appears isointense on T1W and TOF images, and hypointense on T2W images. An *ex vivo *carotid CMR study by Fabiano et al. using these criteria found the sensitivity and specificity for identifying the lipid core according to these criteria were 92% and 74% respectively [43]. A quatitative *in vivo *study by Saam et al. produced a 92% of sensitivity and 65% for specificity comparing the size of the lipid rich necrotic core to corresponding histology [44].

**Figure 4:**
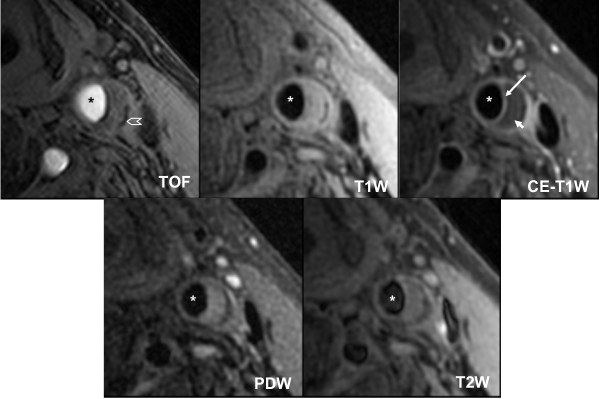
**Atherosclerotic Carotid Artery Lipid-rich Necrotic Core**. Multi contrast black and bright blood sequences show a large lipid-rich necrotic core (small arrow) with an intact thick fibrous cap best seen in the post contrast T1 weighted (CE T1W) image (long arrow). Calcification is also visible at the base of the plaque (chevron). Asterisks are placed on the lumen.

Gadolinium-based contrast agents provided additional information for characterization of the necrotic core and the fibrous cap (Figure 4) [17,18,45]. The addition of contrast agent can increase the signal intensity of fibrous tissue by 79.5% due to the presence of microvasculature; conversely, due to the lack of microvasculature, the necrotic core has the least enhancement 28.6% [17]. The minimal enhancement of the LRNC results in good contrast with fibrous tissue and can be used to determine the boundary of the necrotic core with good accuracy as shown in Figure 4. Wasserman et al. found that gadolinium-enhanced T1W images could delineate the fibrous cap from the LRNC, with a contrast-to-noise ratio similar or better than that of T2W images but with approximately twice the signal-noise-ratio [18]. Takaya et al. also found that *in vivo *CMR measurement of LRNC size was more reproducible when using contrast agent [46]. Cai et al. took contrast one-step further by comparing the size of the enhanced fibrous cap to corresponding histology (For maximal thickness: r = 0.78, *p *< 0.001; for length: r = 0.73, *p *< 0.001; for area: r = 0.73, *p *< 0.001) [45].

#### Intraplaque hemorrhage

Intraplaque hemorrhage is thought to originate primarily from immature neovessels within the plaque that are prone to leakage [47]. The role of intraplaque hemorrhage in plaque development and rupture is not fully understood, but is currently believed to be a driving force in plaque progression through lipid accumulation from red blood cells [48]. It has also been implicated as a risk factor for plaque disruption, although previous studies testing its association with symptoms have varied results [32,49,50].

**Figure 5:**
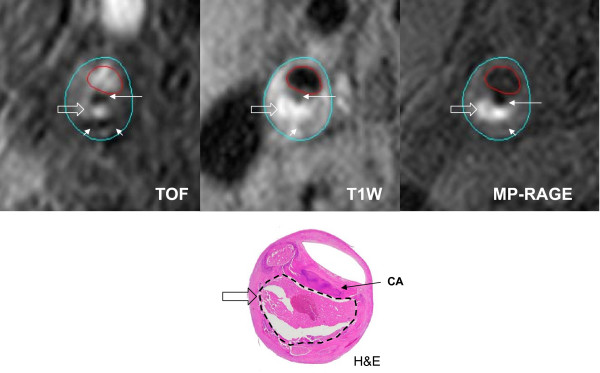
**MR bright-blood sequence Time of Flight (TOF), and black-blood sequences T1W, and MPRAGE show a hyperintense signal (white block arrows) in the necrotic core**. Hematoxylin and Eosin (H&E) stained matching histology shows a large necrotic core (broken lines) filled with hemorrhage. The hypointense signal (thin arrow) in the fibrous cap is shown by matching histology to be an area of calcification.

Intraplaque hemorrhage (Figure 5) is most reliably detected by CMR in the methemoglobin stage due to short T1 relaxation time leading to hyper-intense signal on T1W images. In 2004 Chu et al. used a combined black blood T1 weighted turbo spin echo (TSE) sequence and a time of flight (TOF) sequence for hemorrhage detection. Hemorrhage was identified by hyper-intense signal intensity on both the TOF and T1W images. A T2W black blood TSE sequence was used to stage the early (hypo-intense on T2W images) or later phase (iso- or hyper-intense o) of the methemoglobin phase. Validation with corresponding histology showed that the technique had a sensitivity of 0.85 - 0.95 and specificity of 0.7 - 0.77 [51]. Moody et al. proposed another approach to the identification of intraplaque hemorrhage by using a heavily T1 weighted sequence 3D MP-RAGE (Magnetization-Prepared Rapid Acquisition Gradient-Echo) [16,52]. This technique takes advantage of the short T1 relaxation time of methemoglobin and produces a very bright signal. The technique has a reported sensitivity and specificity of 84% and 84% [52]. The limitation of MP-RAGE is that by using only the T1 shortening effect of methemoglobin, it cannot be used to differentiate the various stages of intraplaque hemorrhage.

#### Calcification

Calcification is commonly found in atherosclerosis and occurs both in areas that are viable and necrotic. Since the role of calcium deposition and its contribution to plaque stability is not well understood, there is a debate as to the extent to which plaque calcification contributes to plaque stability. Some studies demonstrated that the presence and extent of calcification may be associated with increased risk of plaque rupture [53-57], whereas others suggested that it has beneficial effects in stabilizing the plaque, making it stiffer and less prone to rupture [58-60]. There is current growing interest in the location of calcium, which may affect plaque vulnerability. Using a biomechanical model, Li et al. showed that predicted maximum stress was increased by 47.5% when calcium deposits were located in the thin fibrous cap. While the presence of calcium deposits in the lipid core or remote from the fibrous cap resulted in no increase in maximum stress [61,62]. It was conjectured that the presence of calcification within the lipid core may even stabilize the plaque by adding bulk [61]

**Figure 6:**
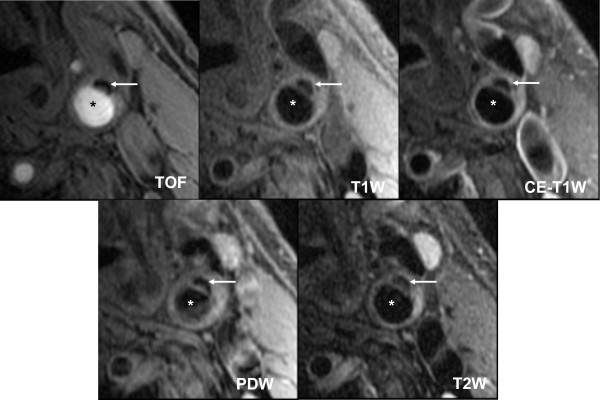
**Calcification can be found in early atherosclerotic lesions**. Black and bright blood multicontrast images show the presence of calcification in the wall of an early lesion of the left common carotid artery (arrow). Asterisks are placed on the lumen.

MR imaging is able to detect plaque calcification with good sensitivity and specificity. Calcification (Figure 6) produces a hypo-intense signal on all contrast weightings due to low water content [63,64]. An 1.5 T CMR investigation by Fabiano et al. demonstrated that CMR has high sensitivity (98%) and specificity (99%) for the identifying plaque calcification [43]. Furthermore, Saam et al. reported a strong correlation between CMR and corresponding histology calcification area measurements (r = 0.74, *p *< 0.001) [44]. At higher field strengths, the increased susceptibility of calcification may alter quantification and/or detection. Underhill et al. compared the quantification of carotid calcification at 1.5 T with those at 3.0 T magnetic resonance imaging, and found that the measurement of calcification was significantly larger at 3.0 T (mean area of 7.9 mm^2 ^vs. 5.9 mm^2^, *p *= 0.03), but a high intraclass correlation of 0.79 was maintained [64].

#### Inflammation

Inflammation is the process by which the body responds to injury. In atherosclerotic disease the injury is the accumulation of lipids in the artery wall. As the lesion evolves proinflammaotry cytokines are expressed. These cytokines initiate a cascade that trigger endothelial cells to express adhesion molecules which then allow for the attachment of blood leucocytes [65]. The infiltration of inflammatory cells into the wall is the hallmark of early atherosclerotic disease and has been implicated in plaque rupture of advanced disease [66]. In the progression of carotid atherosclerosis, inflammation is characterized by increased endothelial permeability, macrophage infiltration, hypoxia within the vessel wall, and growth of the adventitial vasa vasorum. The recruited macrophages consume lipids, undergo apoptosis and thereby contribute to the development and growth of the necrotic core [66].

**Figure 7:**
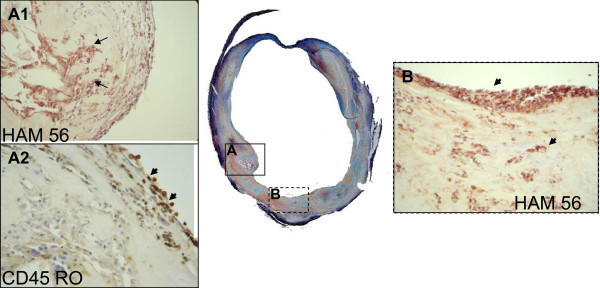
**The Movat's Pentachrome stain of an atherosclerotic common carotid shows that a large lumen can mask complex inflammatorylesions**. Inset **A1 **shows a well developed core surrounded by macrophages stained brown with the macrophage marker HAM 56 (arrows). Inset **A2 **is a high power view of the same lesion stained with the antibody to CD45RO and shows activated T cells on the lumen surface (short arrows). Inset **B **shows macrophages (brown) clustered on the lumen surface (short arrow) and throughout the neointima (short arrow) indicating plaque activity and progression. (Magnification 10× for full figure and 400 × for insets)

Within the atherosclerotic plaque, the distribution of inflammation is also associated with plaque stability (Figure 7). Inflammation is prevalent on the edges of the necrotic core and most importantly in the fibrous cap covering the core [67-71]. As inflammation progresses microvessels are recruited to supply the burgeoning population of cells. Moreno et al. found that microvessel density not only increased with inflammation but also correlated with plaque rupture [72]. Mofidi et al. found a significant association of increased microvasculature and with intraplaque hemorrhage, unstable atherosclerotic lesions and lesions obtained from symptomatic patients [73].

Based on the importance of plaque inflammation in determining the risk of progression or rupture considerable interest exists in the detection of vessel wall inflammation and its precursors. One CMR technique for assessing inflammatory burden in atherosclerosis is dynamic contrast enhanced CMR (DCE-CMR) with kinetic analysis of the contrast agents uptake. DCE-CMR methods, which use clinically available contrast agent, are widely used in cancer studies to assess tissue microvascularity and permeability [74]. In atherosclerotic plaque, microvessels, particularly those with high permeability, are thought to be the primary route for inflammatory cell infiltrate. These vessels also support existing macrophage metabolism. Thus, using kinetic modeling [75], the DCE-CMR parameters describing tissue microvascularity and permeability are considered to be markers of inflammation [76,77].

**Figure 8:**
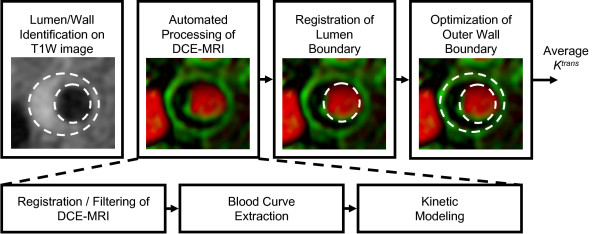
**Contrast Enhanced MRI (DCE-MRI) with Kinetic Remodel**. V-V image generated by the dynamic contrast enhanced MRI (DCE-MRI) with kinetic remodel. Illustration of the steps in creating a vasa vasorum image: boundaries drawn on a conventional contrast define a region of interest for processing. Within this region, DCE-MRI images are run through the KFRS algorithm, and then a blood curve (AIF) is automatically extracted and used for kinetic modeling. Finally, the boundaries are mapped to the resulting vasa vasorum image and Ktrans is measured in the wall. Reprinted, with permission from Ref [77].

A parameter of kinetic modeling derived from the DCE-CMR, fractional plasma volume (v_p_), is associated with a histological assessment of actual microvessel area. A correlation of 0.80 between v_p _measurements and the fractional area of microvessels determined from histology sections was found in 16 patients undergoing CEA, indicating that v_p _was an effective marker of plaque microvasculature [78]. Another parameter of kinetic modeling, the transfer constant (K^trans^) of the contrast agent, estimates the permeability of microvessels. In 27 patients, with analyzable CMR images and corresponding histology obtained from CEA, measurements of K^trans ^were found to correlate with macrophage (r = 0.75, *p *< 0.001) and microvessel (r = 0.71, *p *< 0.001) content [76]. A recent study by Kerwin et al. has shown that K^trans ^measurements of the adventitial region of the carotid were significantly correlated with serum markers of inflammation, such as C-reactive protein levels (r = 0.57; *p *= 0.01) [77]. Figure 8 illustrates the steps necessary to calculate the v_p _and K^trans^, which are represented by red (v_p_) and green (K^trans^) channel in vasa vasorum imaging (V-V image).

DCE-CMR comes with several further advantages. DCE-CMR is easily integrated into existing CMR techniques for vessel wall characterization; it is a short procedure lasting minutes; it utilizes clinically available contrast agents that are safe in people with normal renal function; and the kinetic parameters can be measured with sub-millimeter resolution.

Another contrast-enhanced CMR technique using Ultrasmall Super-Paramagnetic Iron Oxide (USPIO) has been shown to identify carotid inflammatory burden in human studies *in vivo *[79,80]. USPIO particles, which enter atherosclerotic plaques with increased endothelial permeability and accumulate in macrophages, are visualized as a signal dropout or voids on CMR [81]. The accumulation of USPIOs in macrophages is a slow process so that the voids must be identified by comparing images obtained before and, ideally, 36-48 hours after injection [82]. Areas of focal signal loss on CMR images have been shown to correspond to accumulation of macrophage infiltration [83]. In a histological study, Trivedi et al. showed macrophages visualized by USPIO were present in 75% (27/36) of the ruptured or rupture-prone plaques and 7% (1/14) of the stable athermatous lesions [79].

#### Plaque Burden

In addition to the assessment of plaque composition, disruption, and biology, black-blood CMR also permits simple measurements of wall thickness, area, and volume. These measurements are commonly grouped under the name "plaque burden." Measurements of plaque burden may be of particular importance in assessing the early formation of atherosclerotic plaque. It has been conjectured that the initiation of the atherosclerotic lesion begins when low-density lipoprotein (LDL) cholesterol molecules penetrate the arterial wall [37,84,85]. The lipids are taken up by macrophages and become fatty streaks. Due to the normal reactive response to injury (lipids), the arterial wall thickens. Thus, early disease is most readily determined by wall thickness. Historically, the measurement of arterial wall thickness has been performed by B mode ultrasound (B-mode US) as the degree of intima-media thickness (IMT) in the carotid artery. Several studies have been shown that carotid vessel wall CMR can measure arterial wall accurately [86] and reproducibly on multiple scanner platforms [87], between scans and between readers [88-90]. In addition, measurements of the wall thickness by carotid vessel wall CMR have a high correlation with the IMT by B-mode US. Underhill et al. found that a high correlation between measurements of wall thickness on B-mode US and CMR (r = 0.93, *p *< 0.001) [91]. Thus carotid vessel wall CMR is well poised for use in studies of early carotid atherosclerosis.

### Clinical roles for carotid vessel wall CMR

#### Retrospective Risk

To establish the potential for carotid CMR to be used in the assessment of stroke risk, numerous studies have retrospectively investigated the relationship between prior ischemic strokes or transient ischemic attacks and the presence of disrupted plaque. In 23 unilateral symptomatic patients with > 50% stenosis on the symptomatic side, Saam et al. showed an association between symptoms and ruptured plaques when compared to the asymptomatic plaques (78% vs. 30%, *p *= 0.007) [92]. They also found that symptomatic plaques were associated with a higher occurrence of thrombus (61% vs. 26%, *p *= 0.039) [92]. Sadat et al. discovered the utility of multi-contrast CMR while looking at carotid plaques of 60 patients, fibrous cap rupture was observed in 50% of the acute symptomatic patients, 35% of recently symptomatic patients and no fibrous cap rupture was found in asymptomatic patients [93]. Yuan et al. found that patients with ruptured caps were 23 times more likely to have had a recent TIA or stroke, compared with patients with thick fibrous caps [94].

Additional studies have explored the relationship between other features of the carotid plaque and prior symptoms. Murphy et al. found that hemorrhage was occurred significantly more common in the patients' ipsilateral vessels compared with the contralateral, asymptomatic side (60% vs. 36%, *p *< 0.001), and no hemorrhage was found in a gender and age matched healthy control group [95]. Howarth et al. also explored the degree of CMR defined inflammation using USPIO particles, between symptomatic and asymptomatic carotid plaques [96]. They found that symptomatic patients had more focal areas of signal drop than asymptomatic individuals (75% vs. 32%, *p *< 0.01), thus suggesting that symptomatic plaques had large inflammatory infiltrates. Interestingly, asymptomatic individuals more frequently showed signal enhancement, which they attributed to low concentrations of USPIOs achieving positive contrast within thicker fibrous caps. Furthermore, they reported that some asymptomatic plaques also had focal areas of signal drop by using USPIOs, suggesting an occult macrophage burden [96].

#### Prospective Risk

A more compelling argument for carotid CMR risk assessment comes from its ability to prospectively associate CMR features of plaque with future development of symptoms. In a prospective study of 154 subjects with a mean follow-up period of 38 months, Takaya et al. showed that the presence of thin or disrupted fibrous cap, intraplaque hemorrhage, larger lipid-rich necrotic core or thicker wall was associated with an increased occurrence of subsequent neurological complications [97]. In a similar study, Altaf et al. found that intraplaque hemorrhage was a good predictor of ipsilateral stroke and TIA in 64 patients with symptomatic mild to moderate (30%-69%) carotid stenosis over 2 years (hazard ratio = 9.8, *p *= 0.03) [98].

#### Plaque Natural History Studies

CMR has also played a role in understanding what aspects of plaque morphology affect plaque progression and population differences in the disease. These factors may play a role in improved, patient-specific treatment of the disease. In an ethnicity-based study, Saam et al. investigated symptomatic carotid disease in Mainland Chinese patients and a comparable group of Caucasian Americans by using carotid vessel wall CMR [99]. They found significant differences between the Chinese and Americans for the lipid-necrotic core (13.6 vs. 7.8 mm^2^, *p *= 0.002). Also the wall area in the common carotid artery was larger in the Chinese population (52.5 vs. 37.5 mm^2^, *p *= 0.007). In a natural history study, Takaya et al. found that repeated bleeding into the plaque may produce a stimulus for the progression of atherosclerosis by increasing lipid core and plaque volume and creating new destabilizing factors [97]. Hemorrhage into the carotid atherosclerotic plaque accelerated plaque progression in an 18-month period. The change in wall volume (6.8% vs. -0.15%, *p *= 0.009) and lipid-rich necrotic core volume (28.4% vs. -5.2%, *p *= 0.001) was significantly higher in the hemorrhage group than in controls over the course of the study. Furthermore, those with intraplaque hemorrhage at baseline were much more likely to have new plaque hemorrhages at 18 months compared with controls (43% vs. 0%, *p *= 0.006). Besides plaque composition change, in another natural history study, Saam et al. identified factors that may alter the rate of progression in plaque burden [100]. On average, the wall area increased by 2.2% per year (*p *= 0.001). Findings from this study suggested that increased normalized wall index (> 0.64, *p *= 0.001) and the use of statin therapy (*p *= 0.01) were associated with reduced rates of plaque burden progression amongst asymptomatic patients (n = 68) with advanced carotid atherosclerosis (stenosis ≥ 50%) over an 18-month period.

#### Pharmaceutical Trials

The ability of carotid vessel wall CMR to characterize changes in plaque over time has had perhaps its greatest impact in the evaluation of treatment effect in clinical trials. Multiple, long-term, randomized studies have clearly proven that statin therapy reduces clinical events [101-103]. Several prospective CMR investigations have demonstrated that statin therapy regresses carotid plaque burden by using CMR [104,105]. In addition, statin therapy has also been shown to specifically reduce the size of the LRNC [106]. Recently, Zhao et al. reported carotid CMR results from a randomized, double-blind clinical trial directed at assessing the lipid-depletion hypothesis [107]. They found in subjects with a LRNC (n = 33), the LRNC on average decreased by 12 mm^3 ^(p = 0.007) in 1^st ^year, by 13 mm^3 ^(p = 0.004) in 2^nd ^year, and by 0.7 mm^3 ^(p = 0.3) in 3^rd ^year [107]. In addition, in the ORION trial (4522IL/0044), Underhill et al. reported the similar effect of rosuvastatin on LRNC in the patients with moderate hypercholesterolemia, regardless of high-dose (40 mg) or low-dose (5 mg) randomization [106] over the course of 2 years of treatment (% LRNC: an overall reduction from baseline of 41.4% ± 9.6%, *p *= 0.005). In a latter natural history study, Underhill et al. found that statin therapy in general was associated with a halting of plaque progression [108]. In this study, participants on statin therapy did not demonstrate any significant change in plaque burden after 18 months compared to the non-statin group that had a significant increase in plaque size [108]. Interestingly, this study also found that intraplaque hemorrhage may offset the effects of statin therapy [108]. The study suggested intraplaque hemorrhage mitigates the effects of statins, an approach beyond lipid lowering therapy may be necessary to counter the effects of intraplaque hemorrhage.

More recently, there were several CMR based studies to investigate the effects of statin therapy on macrophage activity in carotid atherosclerotic plaques. Carotid Plaque Composition (CPC) study is an ongoing study to investigate the effect of intensive lipid therapy on DCE-CMR defined inflammation [109]. After 1 year of treatment, a statistically significant reduction was found in DCE-CMR defined inflammation (*K*^trans ^from 0.085 ± 0.037 to 0.067 ± 0.028 min^-1^, *p *= 0.02). ATHEROMA was a study undertaken to investigate the effects of low-dose (10 mg) and high-dose (80 mg) atorvastatin on macrophage activity in carotid atherosclerotic plaques using serial USPIO-enhanced CMR [110]. A significant reduction from baseline in USPIO-defined inflammation was observed in the 80-mg group at both 6 weeks (signal intensity change [ΔSI] 0.13, *p *= 0.0003) and at 12 weeks (ΔSI 0.20, *p *< 0.0001). No difference was observed with the 10-mg group. However, several drawbacks stand in the way of the widely clinical application of USPIO-enhanced CMR. One is that at least 2 CMR scans are required before and after infusion of contrast medium. In addition, the calculation of percentage reduction in signal loss needs to be more reproducible since the signal reduction is dependent on reproducibility of coil positioning and on image coregistration before and after USPIO infusion.

#### Systemic Risk

A final area for carotid wall CMR that may be of clinical relevance is the potential to use the carotid artery as a marker of overall systemic disease. Individuals may go on to develop atherosclerosis due to the process of aging. A recent study by Keenan et al., imaged 100 healthy subjects (10 per sex per decade), and found that even in normal subjects the carotid wall volume increased with age [111]. The increase in wall volume correlates negatively with wall distensibility giving rise to a combination of age and pre clinical atherosclerosis related changes. These results are important for further carotid MR studies of early atherosclerosis.

The thickness of carotid arterial wall has correlated well with increased risk factors for atherosclerosis, and the likelihood of future vascular events [112]. Mani et al. stated that a greater wall thickness is prevalent among patients with prior major cardiovascular or cerebrovascular events (MACE) [113]. In their study (n = 195), patients with aged 50 years or older and with at least two risk factors for atherosclerosis were recruited, they found that patients with prior MACE had significantly higher MR wall thickness in carotids and thoracic aorta compared to those without prior MACE (Wall thickness carotids: 1.03 ± 0.03 vs. 0.93 ± 0.03, *p *= 0.001; wall thickness aorta: 1.63 ± 0.10 vs. 1.50 ± 0.04, *p *= 0.009, respectively). CMR provides a new opportunity to investigate carotid disease as a systemic marker of overall cardiovascular health.

## Discussion

In summary, carotid vessel wall CMR appears poised for use in clinical applications and is, in fact, having an indirect clinical impact today through clinical trials involving carotid CMR. Numerous studies support the hypothesis that features of the carotid vessel wall identified with CMR can segregate individuals with similar degrees of stenosis into higher and lower risk categories. Thus, carotid vessel wall CMR is a promising candidate to be an additional diagnostic tool for management of patients with carotid atherosclerosis. The achievements of carotid vessel wall CMR to date strongly indicate the need for a large multi-center trial of its merits for patient management.

Additionally, CMR technology applicable to carotid vessel wall imaging is evolving rapidly. Within the next decade, considerable new techniques are expected to further refine our capabilities in charactering carotid atherosclerosis. Most notably, molecular imaging techniques are being developments for highly specific enhancement of components such as fibrin [114], angiogenic integrins [115], macrophage receptors [116], and lipid-rich areas [117]. Ultimate use of these technologies in human is, however, uncertain. In this review, we addressed only technologies that would be suitable for clinical use immediately if indicated.

Also, the vessel wall imaging capabilities of CMR are not limited to the carotid arteries. Imaging methods developed for carotid artery may be translated to other beds, although each one has to tailor to the local environment. Like carotid arteries, femoral arteries are relatively accessible to surface coils and benefit most from improvements in field-strength and surface coil design [118]. In contrast, owing to their size and complex anatomy, coronary and aortic images are impeded by various factors [119,120]. For example, motion compensation needs to be considered in coronary and aortic arteries. Image quality may be improved by using cardiac and respiratory gating. In addition, blood flow velocities and directions in different arterial beds should be considered as they determine blood suppression efficiency in most black-blood techniques.

In conclusion, CMR of the early and advanced atherosclerotic plaque has shown promise in providing understanding of the biology of the disease and a potential to provide clinical risk assessments for individual carotid atherosclerotic plaques. Multi-contrast CMR is currently in use in clinical trials to determine the effectiveness of new drugs and therapies. The capacity to non-invasively monitor the disease may lead to novel therapeutic targets that are associated with rapid progression or a muted response to conventional treatments. With the continual development of CMR techniques, vessel wall CMR is likely to be widely used for atherosclerosis management clinically in the near future.

## Competing interests

This work was funded by the NIH grant RO1 HL56874, a grant from Pfizer, and an NIH-funded collaboration with VPDiagnostics, Inc. R44 HL070576.

## Authors' contributions

LD, WK, MF, RL, JW, HC, GC, TH and CY drafted the manuscript. All authors read and approved the final manuscript.
